# Emerging and Reemerging Diseases in the World Health Organization (WHO) Eastern Mediterranean Region—Progress, Challenges, and WHO Initiatives

**DOI:** 10.3389/fpubh.2017.00276

**Published:** 2017-10-19

**Authors:** Evans Buliva, Mohamed Elhakim, Nhu Nguyen Tran Minh, Amgad Elkholy, Peter Mala, Abdinasir Abubakar, Sk Md Mamunur Rahman Malik

**Affiliations:** ^1^Regional Office for the Eastern Mediterranean, World Health Organization, Cairo, Egypt

**Keywords:** emerging diseases, reemerging diseases, infectious diseases, emergencies, Eastern Mediterranean

## Abstract

The Eastern Mediterranean Region (EMR) of the World Health Organization (WHO) continues to be a hotspot for emerging and reemerging infectious diseases and the need to prevent, detect, and respond to any infectious diseases that pose a threat to global health security remains a priority. Many risk factors contribute in the emergence and rapid spread of epidemic diseases in the Region including acute and protracted humanitarian emergencies, resulting in fragile health systems, increased population mobility, rapid urbanization, climate change, weak surveillance and limited laboratory diagnostic capacity, and increased human–animal interaction. In EMR, several infectious disease outbreaks were detected, investigated, and rapidly contained over the past 5 years including: yellow fever in Sudan, Middle East respiratory syndrome in Bahrain, Oman, Qatar, Saudi Arabia, United Arab Emirates, and Yemen, cholera in Iraq, avian influenza A (H5N1) infection in Egypt, and dengue fever in Yemen, Sudan, and Pakistan. Dengue fever remains an important public health concern, with at least eight countries in the region being endemic for the disease. The emergence of MERS-CoV in the region in 2012 and its continued transmission currently poses one of the greatest threats. In response to the growing frequency, duration, and scale of disease outbreaks, WHO has worked closely with member states in the areas of improving public health preparedness, surveillance systems, outbreak response, and addressing critical knowledge gaps. A Regional network for experts and technical institutions has been established to facilitate support for international outbreak response. Major challenges are faced as a result of protracted humanitarian crises in the region. Funding gaps, lack of integrated approaches, weak surveillance systems, and absence of comprehensive response plans are other areas of concern. Accelerated efforts are needed by Regional countries, with the continuous support of WHO, to build and maintain a resilient public health system for detection and response to all acute public health events.

## Background

Countries in the Eastern Mediterranean Region (EMR) continue to be hotspots for emerging and reemerging infectious diseases (1). Outbreaks of such diseases have a significant impact on health and economic development in the Region. At least 11 of the 22 countries in the Region have reported epidemics from emerging infectious disease over the past 10 years with the potential for global spread (2). These epidemic threats remain potentially devastating to the social and economic development of the Region, combined with the risk for international spread. The need to prevent, detect, and respond to any infectious disease that poses a persistent threat to global health security remains a national, regional, and international priority (3).

The mission of WHO Health Emergencies (WHE) Program is to build the capacity of Member States to manage health emergency risks and, when national capacities are overwhelmed, to lead and coordinate the international health response to contain outbreaks and to provide effective relief and recovery to affected populations.

The Infectious Hazard Management unit of WHE program in the EMR of World Health Organization (WHO) is responsible for establishing risk mitigation strategies and capacities for priority high-threat infectious hazards. This includes developing and supporting prevention and control strategies, tools and capacities for high-threat infectious hazards, establishing and maintaining experts’ networks to monitor, detect, understand, and manage emerging or reemerging high-threat infectious diseases in the Region.

The principal risk factors contributing to the emergence and rapid spread of epidemic diseases in the Region include acute and protracted humanitarian emergencies resulting in fragile health systems, increased population mobility (travel and displacement), rapid urbanization, climate change, weak surveillance and limited laboratory diagnostic capacity, and increased human–animal interaction (4).

## Current Situation

A summary of recent infectious disease outbreaks in Eastern Mediterranean Region is provided in Table 1. Major infectious disease outbreaks that were detected, investigated and rapidly contained over the past 5 years include yellow fever in Sudan (2012) (849 cases including 171 deaths) (5), Middle East respiratory syndrome in Bahrain, Oman, Qatar, Saudi Arabia, United Arab Emirates and Yemen (2013–2015) (6), cholera in Iraq (2015) (7), avian influenza A (H5N1) infection in Egypt (359 cases including 122 deaths) (8), and dengue fever in Yemen (112 cases including 2 deaths), Sudan (1,151 cases including 9 deaths), and Pakistan (111 cases in 2012-more than 17,000 suspected cases in 2013, 186 cases in 2014) (2012–2015) (9).

**Table 1:** Summary of recent infectious disease outbreaks in Eastern Mediterranean Region.

Outbreak	Country affected and year
Yellow fever	Sudan (2005, 2012, 2013)
MERS-CoV	Bahrain (2016), Egypt (2014), Iran (2014), Jordan (2012), Kuwait (2013), Lebanon (2014, 2017), Oman (2013–2017), Qatar (2012–2017), Saudi Arabia (2012–2017), Tunisia (2013), United Arab Emirates (2013–2017), Yemen (2014)
Cholera	Afghanistan (2012, 2013), Iraq (2012), Somalia (2012, 2016, 2017), Yemen (2016, 2017)
Influenza A (H5N1)	Egypt (2006–2017)
Dengue fever	Djibouti (2012), Egypt (2015), Oman (2014), Pakistan (2012, 2013, 2014, 2017), Yemen (2012, 2016), Sudan (2014)
Chikungunya	Pakistan (2016), Somalia (2016), Yemen (2010)
Crimean–Congo haemorrhagic fever	Afghanistan (2007, 2012, 2017), Iran (2000, 2012), Pakistan (2000, 2012, 2013, 2014, 2017)
Influenza A (H1N1) pdm09	Afghanistan, Bahrain, Egypt, Iran, Iraq, Jordan, Kuwait, Lebanon, Libya, Morocco, Occupied Palestinian territory, Oman, Qatar, Saudi Arabia, Sudan, Syrian Arab Republic, Tunisia, United Arab Emirates, Yemen (2009)

Dengue fever remains an emerging public health concern in the region (9). Countries from the Red Sea rim frequently report either sporadic cases, or explosive outbreaks during high transmission season (10). At least eight countries in the Region are endemic for dengue, with a high abundance of competent vectors such as *Aedes* mosquitoes (9). Outbreaks have been reported from Egypt (2015) (9), Pakistan (2011 and 2014) (11), Sudan, and Yemen (2012–2015) (9), and imported cases in Oman (2014 and 2015) (12). Djibouti and Somalia have reported sporadic cases signifying presence of competent vectors (13). The principal risk factors for dengue cases transmission in the Region include increased urbanization, uncontrolled population growth in urban and peri-urban areas, unprecedented increase in travel by air as well as poor vector control intervention (14).

As of June 2016, none of the countries in the EMR had reported Zika virus infection but the risk remains considerable (15). This risk is highest in eight countries, Djibouti, Egypt, Oman, Pakistan, Saudi Arabia, Somalia, Sudan, and Yemen, where *Aedes aegypti* mosquitoes exist (16). Furthermore, the invasive mosquito *Aedes albopictus*, another vector of mosquito-borne disease, is spreading in the Region and has been recently reported in the Islamic Republic of Iran, Lebanon, Palestine, and Syrian Arab Republic (17).

Chikungunya is prevalent in Yemen (1,657 cases), Sudan, and Somalia (18, 19). Yemen faced two major outbreaks in 2011 and 2012 (20, 21), and sporadic cases have been reported from Sudan and Somalia (18, 19).

Sudan remains the only country in the Region with reports of major outbreaks of yellow fever (5). In both 2012 and 2013, the country reported an outbreak with high morbidity and mortality (22). In Somalia and Djibouti, Serological studies show evidence of circulation of yellow fever in Djibouti and Somalia (23).

The emergence of MERS-CoV in the Region and its continuing transmission since 2012 currently poses one of the biggest threats to global health security (24). Most cases (over 85%) reported to date have been from the countries of the Region, notably from Saudi Arabia (1,527 including 624 deaths) (more than 95% of cases), Jordan, Kuwait, Oman (9 cases including 3 deaths), Qatar (16 cases including 4 deaths), and the United Arab Emirates (79 cases including 12 deaths) (25). Laboratory-confirmed human infections with history of travel to one of these countries have also been reported from other countries including Egypt, Islamic Republic of Iran, Lebanon, Tunisia, and Yemen (1 death), bringing the total number of countries reporting laboratory-confirmed cases of MERS-CoV to 12 in the Region (25).

Cholera reemerged in the Region in 2015 (26). Afghanistan, Pakistan, and Somalia, the three most cholera-endemic countries in the Region, continue to report sporadic cases (27). Imported cases of cholera were reported in 2015 from Bahrain, Islamic of Iran Kuwait Lebanon, Oman, and Qatar (26). An outbreak was reported from Iraq (2,810 cases including 2 deaths) after a gap of nearly 3 years (28). The outbreak threatened to spill over to other countries owing to frequent cross-border movement between Iraq and other neighboring countries affected by conflict (7). Available surveillance data show that nine countries in the Region are endemic for cholera (29). The potential risk of cholera is heightened in countries hosting a high number of refugees and displaced populations (30).

Crimean–Congo haemorrhagic fever (CCHF), a tick-borne viral infection, continues to cause human infections in the endemic belt of the Region that includes Pakistan (31), Afghanistan, and the Islamic Republic of Iran (32). Recently, the CCHF virus has been found in Oman, which is a sign that this emerging virus may spread to other non-endemic countries (33).

Over the past 5 years, a seasonal surge of influenza caused by influenza A (H1N1) pdm09 was reported from Egypt, Islamic Republic of Iran, Iraq, Jordan, Kuwait, Libya, and Tunisia (34). Egypt, currently, has the highest number of human infections caused by avian influenza A (H5N1) globally and second-highest mortality among all countries affected by H5N1 globally (8). Egypt, last year, also reported two human infections caused by influenza A (H9N2) which are enzootic in poultry populations in parts of the Region (35).

## Progress to Date

A WHO Regional strategy for surveillance, outbreak response, social mobilization, and vector control has been rolled out following a subregional meeting held in 2012 on the control of dengue fever in the Red Sea rim (36). Additionally, the Regional Office deployed staff and international experts to contain outbreaks of dengue in Pakistan during 2012 and 2014 (37), Sudan during 2013 and 2015 (38), and in Yemen during 2012, 2013 and in 2015 (39). WHO also supported training for participants from Djibouti, Saudi Arabia, Somalia, Sudan, and Yemen in the areas of epidemiological surveillance, entomological surveillance and laboratory diagnosis at the Environmental Health Institute of Singapore, which is a WHO collaborating center for reference and research of arbovirus and its associated vectors (40).

Following the 1st February 2016 WHO declaration of a public health emergency of international concern with regard to clusters of microcephaly and neurological disorders potentially associated with Zika virus (41). The Regional Office convened Member States and key partner agencies and developed a range of integrated activities covering prevention, surveillance, and control interventions for *Aedes* mosquitoes in the Region (42).

Sudan launched its first ever yellow fever mass preventive vaccination campaigns in December 2014 vaccinating close to 7.5 million people aged between 9 months and 60 years in seven high-risk states in the country (43). The vaccination campaign was preceded by a risk assessment combining a serological prevalence survey and entomological studies conducted in 2012–2013 covering four ecological zones. Areas with active circulation of yellow fever virus were targeted for preventive vaccination campaigns (44).

Since the emergence of MERS-CoV, WHO has worked closely with the affected Member States in three main areas: improving public health preparedness; outbreak response; and addressing critical knowledge gaps to better understand the epidemiology and transmission patterns of the disease caused by the virus (45). In order to improve detection and response capacity for this novel virus, surveillance efforts were intensified across all countries in the Region (24). WHO is working closely with other international agencies to come up with an appropriate research agenda for both animal and human health to bridge critical gaps (46).

A strategic framework for cholera preparedness and response has been developed to guide Member States in developing country-specific preparedness and response plans (47). Efforts to strengthen the epidemic response capacity included enhancing disease surveillance, improving case management through training of health workers and provision of medical supplies, and deployment of experts to support health authorities and partners. Regional efforts to control and prevent cholera outbreaks entered a new era following the implementation of the first mass vaccination campaign with oral cholera vaccine from the global stockpile in response to the outbreak in Iraq (48).

Surveillance systems for influenza-like illness and severe acute respiratory infections were established in 16 countries in the Region to build local capacity for early detection, recognition, and response to any novel influenza virus with pandemic potential (34). A total of 16 national influenza centers have been established in the Region for influenza virus isolation, sequencing, and antiviral resistance testing (49). In addition, the Pandemic Influenza Preparedness Framework, a unique public–private partnership initiative, was rolled out in seven middle-income and low-income countries with a view to strengthening capacities for detection and response to influenza with pandemic potential and increasing access to vaccines and other pandemic-related supplies (34).

In view of the geographic expansion of CCHF, the Regional Office is scaling up efforts aimed at the prevention and control of human infections, particularly in the areas of surveillance for early detection of human cases, therapeutic options and tick control (50).

A regional network for experts and technical institutions has been established to facilitate support for international outbreak response in response to the growing frequency, duration, and scale of disease outbreaks in the Region (51).

## Challenges

More countries in the Region are currently experiencing protracted humanitarian emergencies, with over 56 million people currently affected (52). These internally displaced persons, refugees, and surrounding host communities are at high risk of the potential outbreaks from infectious disease (53). Epidemic threats are often exacerbated in such situations, owing to fragile public health systems and weakened or fragmented surveillance and threat detection capacities (54).

Regional preparedness and control efforts for dengue fever and other arboviral infections face major challenges such as poor vector surveillance capacities, weak multidisciplinary and inter-sectoral collaboration, lack of integrated vector management approaches, and low risk perception of these infections. Significant funding gaps persist for the prevention and control of arboviral infections (15). Other challenges include knowledge gaps on the risk factors for transmission of emerging infections, weak and variable surveillance systems for early detection and response, limited laboratory diagnostic capacities, insufficient investment in disease surveillance and response activities, and absence comprehensive preparedness and response plans (55).

## Recommended Actions

World Health Organization will continue to support countries in the areas of surveillance, early detection, and response to emerging infectious disease outbreaks. Accelerated efforts are needed by countries to build and maintain a resilient public health system for detection and response to all acute public health events. Countries need to roll out the strategic framework for prevention and control of emerging diseases and develop a framework for integrating the early warning system for disease outbreaks in countries affected by humanitarian crises within the routine disease surveillance system. The IHR (2005) remains the key driver in national and international efforts to strengthen national and global health security.

## Author Contributions

All authors contributed equally in the manuscript.

## Disclaimer

Information in this mini review was obtained from information sources that are publicly available. This information presents the point of view of all contributing authors and not the point of view of the organization (WHO).

## Conflict of Interest Statement

The authors declare that the research was conducted in the absence of any commercial or financial relationships that could be construed as a potential conflict of interest.

## References

[1] Haq Z Mahjour J Khan W. Communicable diseases in the Eastern Mediterranean Region: prevention and control 2010–2011. East Mediterr Health J (2013) 19(10):888–91. 24313154

[2] Abubakar A Malik M Pebody RG Elkholy AA Khan W Bellos A Burden of acute respiratory disease of epidemic and pandemic potential in the WHO Eastern Mediterranean Region: a literature review. East Mediterr Health J (2016) 22(7):513–26. 27714746

[3] Morens DM Fauci AS Emerging infectious diseases: threats to human health and global stability. PLoS Pathog (2013) 9(7):e1003467 10.1371/journal.ppat.1003467 23853589 PMC3701702

[4] Mokdad AH Forouzanfar MH Daoud F El Bcheraoui C Moradi-Lakeh M Khalil I Health in times of uncertainty in the eastern Mediterranean region, 1990–2013: a systematic analysis for the Global Burden of Disease Study 2013. Lancet Glob Health (2016) 4(10):e704–13. 10.1016/S2214-109X(16)30168-1 27568068 PMC6660972

[5] Soghaier MA Hagar A Abbas MA Elmangory MM Eltahir KM Sall AA. Yellow fever outbreak in Darfur, Sudan in October 2012; the initial outbreak investigation report. J Infect Public Health (2013) 6(5):370–6. 10.1016/j.jiph.2013.04.007 23999341

[6] Mohd HA Al-Tawfiq JA Memish ZA. Middle East Respiratory Syndrome Coronavirus (MERS-CoV) origin and animal reservoir. Virol J (2016) 13(1):87. 10.1186/s12985-016-0544-0 27255185 PMC4891877

[7] Mukhopadhyay AK Al Benwan K Samanta P Chowdhury G Albert MJ Vibrio cholerae O1 imported from Iraq to Kuwait, 2015. Emerg Infect Dis (2016) 22(9):1693–4. 10.3201/eid2209.160811 27532267 PMC4994364

[8] Kayali G Kandeil A El-Shesheny R Kayed AS Maatouq AM Cai Z Avian influenza A(H5N1) virus in Egypt. Emerg Infect Dis (2016) 22(3):379–88. 10.3201/eid2203.150593 26886164 PMC4766899

[9] Humphrey JM Cleton NB Reusken CB Glesby MJ Koopmans MP Abu-Raddad LJ. Dengue in the Middle East and North Africa: a systematic review. PLoS Negl Trop Dis (2016) 10(12):e0005194. 10.1371/journal.pntd.0005194 27926925 PMC5142774

[10] Seidahmed OM Hassan SA Soghaier MA Siam HA Ahmed FT Elkarsany MM Spatial and temporal patterns of dengue transmission along a Red Sea coastline: a longitudinal entomological and serological survey in Port Sudan city. PLoS Negl Trop Dis (2012) 6(9):e1821. 10.1371/journal.pntd.0001821 23029582 PMC3459851

[11] Raza FA Rehman S Khalid R Ahmad J Ashraf S Iqbal M Demographic and clinico-epidemiological features of dengue fever in Faisalabad, Pakistan. PLoS One (2014) 9(3):e89868. 10.1371/journal.pone.0089868 24595236 PMC3940608

[12] Al Awaidy ST Al Obeidani I Bawikar S Al Mahrouqi S Al Busaidy SS Al Baqlani S Dengue epidemiological trend in Oman: a 13-year national surveillance and strategic proposition of imported cases. Trop Doct (2014) 44(4):190–5. 10.1177/0049475514541650 24994569

[13] Baba M Villinger J Masiga DK. Repetitive dengue outbreaks in East Africa: a proposed phased mitigation approach may reduce its impact. Rev Med Virol (2016) 26(3):183–96. 10.1002/rmv.1877 26922851

[14] Murray NE Quam MB Wilder-Smith A. Epidemiology of dengue: past, present and future prospects. Clin Epidemiol (2013) 5:299–309. 10.2147/CLEP.S34440 23990732 PMC3753061

[15] Minh NN Huda Q Asghar H Samhouri D Abubakar A Barwa C Zika virus: no cases in the Eastern Mediterranean Region but concerns remain. East Mediterr Health J (2016) 22(5):350–5. 27553402 10.26719/2016.22.5.350

[16] Escadafal C Gaayeb L Riccardo F Pérez-Ramírez E Picard M Dente MG Risk of zika virus transmission in the Euro-Mediterranean area and the added value of building preparedness to arboviral threats from a one health perspective. BMC Public Health (2016) 16(1):1219. 10.1186/s12889-016-3831-1 27914465 PMC5135781

[17] Akiner MM Demirci B Babuadze G Robert V Schaffner F Spread of the invasive mosquitoes *Aedes aegypti* and *Aedes albopictus* in the black sea region increases risk of chikungunya, dengue, and zika outbreaks in Europe. PLoS Negl Trop Dis (2016) 10(4):e0004664 10.1371/journal.pntd.0004664 27115737 PMC4846050

[18] Adam A Seidahmed OM Weber C Schnierle B Schmidt-Chanasit J Reiche S Low seroprevalence indicates vulnerability of Eastern and Central Sudan to infection with chikungunya virus. Vector Borne Zoonotic Dis (2016) 16(4):290–1. 10.1089/vbz.2015.1897 26974266

[19] Zammarchi L Fortuna C Venturi G Rinaldi F Capobianco T Remoli ME Recent chikungunya virus infection in 2 travelers returning from Mogadishu, Somalia, to Italy, 2016. Emerg Infect Dis (2016) 22(11):2025–7. 10.3201/eid2211.161225 27513985 PMC5088032

[20] Malik MR Mnzava A Mohareb E Zayed A Al Kohlani A Thabet AA Chikungunya outbreak in Al-Hudaydah, Yemen, 2011: epidemiological characterization and key lessons learned for early detection and control. J Epidemiol Glob Health (2014) 4(3):203–11. 10.1016/j.jegh.2014.01.004 25107656 PMC7333817

[21] Thabet AAK Al-Eryani SMA Aziz NA Obadi M Saleh M Epidemiological characterization of chikungunya outbreak in Lahj Governorate, Southern Yemen. J Community Med Health Educ (2013) 3:247 10.4172/2161-0711.1000247

[22] Markoff L Yellow fever outbreak in Sudan. N Engl J Med (2013) 368(8):689–91. 10.1056/NEJMp1300772 23387798

[23] Andayi F Charrel RN Kieffer A Richet H Pastorino B Leparc-Goffart I A sero-epidemiological study of arboviral fevers in Djibouti, Horn of Africa. PLoS Negl Trop Dis (2014) 8(12):e3299. 10.1371/journal.pntd.0003299 25502692 PMC4263616

[24] Malik M Elkholy AA Khan W Hassounah S Abubakar A Minh NT Middle East respiratory syndrome coronavirus: current knowledge and future considerations. East Mediterr Health J (2016) 22(7):537–46. 27714748

[25] World Health Organization, Eastern Mediterranean Regional Office. MERS Situation Updates, November–December 2016. (2017). Available from: http://applications.emro.who.int/dsaf/EMROPub_2016_EN_19348.pdf?ua=1

[26] World Health Organization, Eastern Mediterranean Regional Office. Weekly Epidemiological Monitor. (Vol. 8). (2015). 51 p. Available from: http://applications.emro.who.int/dsaf/epi/2015/Epi_Monitor_2015_8_51.pdf

[27] World Health Organization. Weekly Epidemiological Record. (Vol. 91). (2016). p. 433–40. No 38 Available from: http://apps.who.int/iris/bitstream/10665/250142/1/WER9138.pdf?ua=127665620

[28] Shrivastava S Shrivastava P Ramasamy J Successful containment of the 2015 cholera outbreak in Iraq. Community Acquir Infect [Letter to Editor] (2016) 3(1):28–9. 10.4103/2225-6482.179235

[29] Ali M Lopez AL You YA Kim YE Sah B Maskery B The global burden of cholera. Bull World Health Organ (2012) 90(3):209A–18A. 10.2471/BLT.11.093427 22461716 PMC3314202

[30] Sparrow A Almilaji K Tajaldin B Teodoro N Langton P Cholera in the time of war: implications of weak surveillance in Syria for the WHO’s preparedness – a comparison of two monitoring systems. BMJ Glob Health (2016) 1(3):e000029 10.1136/bmjgh-2016-000029 PMC5321343 28588951

[31] Ahmad A Khan MU Crimean-Congo hemorrhagic fever in Pakistan: are we going in the right direction? J Res Pharm Pract (2015) 4(4):173–4. 10.4103/2279-042X.167052 26645021 PMC4645127

[32] Messina JP Pigott DM Golding N Duda KA Brownstein JS Weiss DJ The global distribution of Crimean-Congo hemorrhagic fever. Trans R Soc Trop Med Hyg (2015) 109(8):503–13. 10.1093/trstmh/trv050 26142451 PMC4501401

[33] Al-Zadjali M Al-Hashim H Al-Ghilani M Balkhiar A A case of Crimean-Congo hemorrhagic fever in Oman. Oman Med J (2013) 28(3):210–2. 10.5001/omj.2013.57 23772290 PMC3680643

[34] Malik M Mahjour J Khan W Alwan A Influenza in the Eastern Mediterranean Region: identifying the unknowns for detection and control of epidemic and pandemic threats. East Mediterr Health J (2016) 22(7):428–9. 27714734

[35] Young SG Carrel M Malanson GP Ali MA Kayali G. Predicting avian influenza co-infection with H5N1 and H9N2 in Northern Egypt. Int J Environ Res Public Health (2016) 13(9):886. 10.3390/ijerph13090886 27608035 PMC5036719

[36] World Health Organization, Eastern Mediterranean Regional Office. Report on the Subregional Meeting on Dengue Fever in the Red Sea Rim, July 2011. (2017). Available from: http://applications.emro.who.int/docs/IC_Meet_Rep_2012_EN_14470.pdf

[37] World Health Organization, Eastern Mediterranean Regional Office. In Focus: WHO Support to Pakistan on Dengue Fever. (2017). Available from: http://www.emro.who.int/pak/pakistan-infocus/world-health-day.html

[38] World Health Organization, Eastern Mediterranean Regional Office. News: Dengue Fever Outbreak in Red Sea State, Sudan. (2017). Available from: http://www.emro.who.int/sdn/sudan-news/sudan-dengue-outbreak2014.html

[39] World Health Organization, Eastern Mediterranean Regional Office. WHO Health Emergencies: WHO Scales Up Response to Control Dengue Fever in Yemen. (2017). Available from: http://www.emro.who.int/eha/news/who-scales-up-response-to-control-dengue-fever-in-yemen.html

[40] Environmental Health Institute (EHI) of Singapore. (2017). Available from: http://www.nea.gov.sg/public-health/environmental-public-health-research

[41] World Health Organization. WHO Statement on the First Meeting of the International Health Regulations (2005) (IHR 2005) Emergency Committee on Zika Virus and Observed Increase in Neurological Disorders and Neonatal Malformations. (2017). Available from: http://www.who.int/mediacentre/news/statements/2016/1st-emergency-committee-zika/en/

[42] World Health Organization, Eastern Mediterranean Regional Office. Summary Report on the Regional Meeting to Enhance Preparedness and Response Capacities to Zika Virus Infection; Cairo, Egypt (Round 1) 22–23 February 2016 – Casablanca, Morocco (Round 2) 28–29 February 2016. (2017). Available from: http://applications.emro.who.int/docs/IC_Meet_Rep_2016_EN_16740.pdf?ua=1

[43] reliefweb. Yellow Fever Vaccination Campaign Completed in Sudan. (2017). Available from: http://reliefweb.int/report/sudan/yellow-fever-vaccination-campaign-completed-sudan

[44] World Health Organization, Eastern Mediterranean Regional Office. Weekly Epidemiological Monitor. (Vol. 7). (2014). Available from: http://applications.emro.who.int/dsaf/epi/2014/Epi_Monitor_2014_7_47_48.pdf

[45] Malik MR Mafi AR Mahjour J Opoka M Elhakim M Muntasir MO. Novel coronavirus infection in the eastern mediterranean region: time to act. East Mediterr Health J (2013) 19(Suppl 1):S31–8. 23888793

[46] World Health Organization, Eastern Mediterranean Regional Office. News: WHO’s High-Level Mission to Saudi Arabia on Middle East Respiratory Syndrome Coronavirus (MERS-CoV) 11–14 January 2016. (2017). Available from: http://www.emro.who.int/surveillance-forecasting-response/surveillance-news/mers-mission-january2016.html

[47] World Health Organization, Eastern Mediterranean Regional Office. Summary Report on the Consultative Meeting on a Strategic Approach for Cholera Preparedness and Response in the Eastern Mediterranean Region; Amman, Jordan 17–19 November 2015. (2017). Available from: http://applications.emro.who.int/docs/IC_Meet_Rep_2016_EN_18674.pdf?ua=1

[48] Lam E Al-Tamimi W Russell SP Butt MO Blanton C Musani AS Oral cholera vaccine coverage during an outbreak and humanitarian crisis, Iraq, 2015. Emerg Infect Dis (2017) 23(1):38–45. 10.3201/eid2301.160881 27983502 PMC5176248

[49] World Health Organization, Eastern Mediterranean Regional Office. News: Influenza Surveillance in the Region. (2017). Available from: http://www.emro.who.int/surveillance-forecasting-response/surveillance-news/influenza-surveillance-in-the-region.html

[50] World Health Organization, Eastern Mediterranean Regional Office. Summary Report on the Meeting on Prevention and Control of Crimean–Congo Haemorrhagic Fever in the Eastern Mediterranean Region; Muscat, Oman 7–9 December 2015. (2017). Available from: http://applications.emro.who.int/docs/IC_Meet_Rep_2016_EN_18675.pdf?ua=1

[51] World Health Organization, Eastern Mediterranean Regional Office. Summary Report on the Meeting to Establish a Regional Network for Outbreak Alert and Response; Casablanca, Morocco; 19–21 October 2015. (2017). Available from: http://applications.emro.who.int/docs/IC_Meet_Rep_2016_EN_18673.pdf

[52] Aziz NA Managing migration in the Eastern Mediterranean: challenges and opportunities. IEMed Mediterranean Yearbook 2016, Cairo: American University in Cairo (2016). p. 109–12.

[53] Seita A. Complex emergencies in the Eastern Mediterranean region: impact on tuberculosis control. Int J Mycobacteriol (2016) 5(Suppl 1):S12. 10.1016/j.ijmyco.2016.10.040 28043499

[54] Santoro A Abu-Rmeileh N Khader A Seita A McKee M. Primary healthcare reform in the United Nations Relief and Works Agency for Palestine Refugees in the Near East. East Mediterr Health J (2016) 22(6):417–21. 27686983 10.26719/2016.22.6.417

[55] Malik MR Mahjour J Preparedness for Ebola: can it transform our current public health system? East Mediterr Health J (2016) 22(8):566–7. 27834437

